# The Biology of the Cytolethal Distending Toxins

**DOI:** 10.3390/toxins3030172

**Published:** 2011-03-07

**Authors:** Lina Guerra, Ximena Cortes-Bratti, Riccardo Guidi, Teresa Frisan

**Affiliations:** Department of Cell and Molecular Biology, Karolinska Institute, Stockholm, Sweden, Box 285, S-171 77 Stockholm, Sweden; Email: lina.guerra@ki.se (L.G.); ximena.cortes.bratti@ki.se (X.C.-B.); riccardo.guidi@ki.se (R.G.)

**Keywords:** cytolethal distending toxin, bacterial genotoxin, toxin internalization, DNA damage, DNA damage response, survival signals, virulence factor, chancroid, periodontitis, colitis/hepatitis

## Abstract

The cytolethal distending toxins (CDTs), produced by a variety of Gram-negative pathogenic bacteria, are the first bacterial genotoxins described, since they cause DNA damage in the target cells. CDT is an A-B_2_ toxin, where the CdtA and CdtC subunits are required to mediate the binding on the surface of the target cells, allowing internalization of the active CdtB subunit, which is functionally homologous to the mammalian deoxyribonuclease I. The nature of the surface receptor is still poorly characterized, however binding of CDT requires intact lipid rafts, and its internalization occurs via dynamin-dependent endocytosis. The toxin is retrograde transported through the Golgi complex and the endoplasmic reticulum, and subsequently translocated into the nuclear compartment, where it exerts the toxic activity. Cellular intoxication induces DNA damage and activation of the DNA damage responses, which results in arrest of the target cells in the G1 and/or G2 phases of the cell cycle and activation of DNA repair mechanisms. Cells that fail to repair the damage will senesce or undergo apoptosis. This review will focus on the well-characterized aspects of the CDT biology and discuss the questions that still remain unanswered.

## 1. Introduction

The cytolethal distending toxins (CDTs) comprise a family of bacterial proteins toxins produced by a variety of Gram negative bacteria, such as *Escherichia coli*, *Aggregatibacter actinomycetemcomitans*, *Haemophilus ducreyi*, *Shigella dysenteriae*, *Campylobacter* sp., *Helicobacter* sp., and *Salmonella enterica*.

CDT and Colibactin, a putative hybrid peptide-polyketide genotoxin produced by commensal *E. coli* strains [1], are the first bacterial genotoxins described, having the unique characteristic to cause DNA damage in the target cells.

In this review, we will focus on the molecular mode of action, the internalization pathway and the cellular responses induced by CDT intoxication. We will further discuss the role of these toxins as virulence factors in bacterial pathogenesis.

To facilitate the reading, we have adopted the nomenclature proposed by Thelestam *et al.*, where each CDT is specified by indicating the initials of the producing bacterium before CDT and, if necessary, the strain number or other common designation after CDT (e.g., HdCDT: *H. ducreyi* CDT or EcCDT-I: *E.* *coli* CDT type I) [2].

## 2. CDT Structure and Enzymatic Activity

CDT is the product of an operon encoding three proteins: CdtA, CdtB and CdtC. All three subunits are essential to confer full activity of the holotoxin (reviewed in [3]).

The crystal structure of the *H. ducreyi* CDT (HdCDT) has been solved by Nesic and collaborators, and revealed that the holotoxin is a tripartite complex. The CdtA and CdtC subunits are lectin-type molecules, sharing structural homology with the B-chain repeats of the plant toxin ricin. The CdtB subunit adopts the canonical four-layered fold of the DNase I family: a central 12-stranded β-sandwich packed between outer α-helices and loops on each side of the sandwich [4]. The crystal structure confirms previous data, demonstrating that CdtB shares five conserved residues with the active site of the mammalian DNase I, and possesses DNase capacity *in vitro* and when ectopically expressed or microinjected in eukaryotic cells. Mutation in any conserved residue important for the catalytic activity or the Mg^2+^ binding abolishes the ability of CdtB to cleave DNA *in vitro* and to induce DNA damage responses *in vivo* [5,6,7,8].

The three subunits form a complex with three globular protein-protein interfaces (CdtA-CdtB, CdtA-CdtC and CdtB-CdtC). Furthermore, the CdtA and CdtC subunits present non-globular amino acid extensions at the amino- and carboxyl-termini, which interact with each other and with the CdtB subunit. Two very conserved structures can be observed within the surface formed by the CdtA and CdtC subunits: (1) a large aromatic cluster of eight bulky side-chains in CdtA; (2) a deep groove formed by the juxtaposition of these subunits. Mutations of the aromatic patch do not change the stability of the ternary complex, but completely abolished the ability of the toxin to cause cell cycle arrest in the human cell line HeLa, suggesting that it plays a relevant role in modulating toxin binding to its receptor [4].

The CdtB subunit is the most conserved component of the holotoxin amongst all the CDT-producing bacteria. The overall sequence identities of CdtA and CdtC homologs are generally less than 30%. However modeling studies based on the HdCDT crystal structure showed that a number of structural features are remarkably conserved, such as the close interplay of the CdtA and CdtC subunits in the formation of the groove and aromatic patch, and the similarity in their positioning with the two lectin repeats in the ricin B-chain. This suggests that these two components of CDT work together to mediate cell surface binding and internalization of the holotoxin [9].

Based on these data, CDT can be regarded as an A-B_2_ toxin, where CdtA and CdtC are required for binding the holotoxin to the plasma membrane of the target cells, allowing entry of the active CdtB, which can translocate to the nucleus and induce DNA lesions.

There are still several open questions regarding the interaction of the holotoxin with the target cells. Little information is available on the biogenesis of CDT holotoxin. Furthermore, it is still not clear how CdtA and CdtC contribute to the binding on the plasma membrane, and the nature of the CDT receptor still remains unknown.

To study the biogenesis of A. *actinomycetemcomitans* (AaCDT), Ueno and co-workers have used an *E. coli* strain carrying the *A. actinomycetemcomitans cdtABC* genes. They have shown that membrane-associated CdtA is a lipoprotein. In the periplasm, CDT is a complex composed of CdtA, CdtB, and CdtC, whereas CDT in the culture supernatant contains an *N*-terminally truncated CdtA. This suggests that CdtA undergoes lipid modification during the export process and subsequent *N*-terminal processing after forming a complex with CdtB and CdtC in the periplasm [10].

Using enzyme-linked immunosorbent assays or FACS analysis it was shown that the CdtA and CdtC subunit from the *Campylobacter jejuni* (CjCDT), *E. coli* (EcCDT-II), and AaCDT bind with specificity to the surface of the human cell lines HeLa [11,12] or U937 [13], whereas CdtB does not. Conversely, Di Rienzo and colleagues showed that the *A. actinomycetemcomitans* CdtA, but not the CdtC subunit, binds to the surface of Chinese hamster ovary (CHO) cells [14]. This discrepancy could be related to the different profile of surface molecules expressed on the cell types used. Alternatively, it is possible that the CdtA subunit is responsible to bind on the surface of the plasma cell, while CdtC may assist the trafficking of the CdtB subunit toward the nuclear compartment, as demonstrated for the AaCDT [15]. This hypothesis is supported by the fact that the CdtC, but not the CdtA subunit, transits via the Golgi complex similarly to the active CdtB component of the holotoxin [16].

What is the nature of the CDT receptor? Several studies has been conducted, and yielded to contrasting results.

EcCdtA-II and CjCdtA are able to compete with the binding of the corresponding CdtC subunits, indicating that they interact with the same structure on the target cell, which was identified as *N*-linked glycoproteins [12]. Moreover, EcCDT binds fucose *in vitro*, and fucose-specific lectins block Ec-CDT-mediated cell cycle arrest, presumably by preventing binding of toxin to its receptor. These findings suggested that fucose is a binding determinant for Ec-CDT.

Another study indicated that, similarly to other bacterial proteins toxins, the CDT holotoxin binds to surface glycosphingolipids. Inhibitors of glycosphingolipids synthesis prevents intoxication of the human monocytic U937 cell line by AaCDT, and thin layer chromatography demonstrates a strong binding of the CdtA with GM1, GM2, GM3, and Gb4, while CdtC reacts strongly with GM1 and GM2. Intoxication of the U937 cell line is blocked by preincubation of toxin with liposomes that contain GM3 [13].

Using laser confocal microscopy Shenker *et al.*, have demonstrated that the AaCDT colocalizes with GM1-enriched membrane regions of the plasma membrane, which are characteristic of membrane rafts [8,17]. Cholesterol depletion by methyl β-cyclodextrin reduces the ability of the AaCDT or HdCDT to associate with Jurkat or HeLa cell lines, respectively, and prevents the toxic activity [8,17].

Site directed mutagenesis of a human cell line haploid for all chromosomes except chromosome 8 identified SGMS1 and TMEM181 mutants as resistant to EcCDT. The SGMS1 mutation reduces levels of sphingomyelin, a key component of lipid rafts, confirming the previous data obtained for HdCDT and AaCDT. TMEM181 is present at the cell surface. Flag-tagged CDT is able to bind HA-tagged TMEM181, suggesting that this protein may be the EcCDT receptor, however it could not be ruled out that TMEM181 plays a role in the trafficking of the receptor-toxin complex [18].

The divergent results on factors that mediate the toxin binding on the cell surface may depend on the different receptor specificity exhibited by each CDT. It is noteworthy that several CDT are species specific: HdCDT and AaCDT cannot intoxicate cells of rodent origin. Furthermore, Eshraghi and co-workers have demonstrated that CDTs from *H. ducreyi*, *A. actinomycetemcomitans*, *E. coli*, and *C. jejuni* differ in their abilities to intoxicate host cells. Binding of Aa, Hd, and EcCDT-III, but not CjCDT is dependent on the presence of cholesterol. Surprisingly, mutant CHO cells that lack *N*-linked complex and hybrid carbohydrates, cells that lack glycosphingolipids or are deficient in fucose biosynthesis are similarly sensitive as the wild type to intoxication by all four CDTs tested, indicating that *N*- and *O*-glycan, or fucosylated structures are dispensable to mediate toxin binding [19].

An exception to the general structure of the CDT holotoxin is the CDT produced by *S. enterica*, serovar Typhi CT18. The *cdtB* gene is located within a region of the chromosome, however there are no homologues of the *cdtA* or *cdtC* genes [20]. Ectopic expression of the StCdtB in Cos-2 is sufficient to cause DNA damage, and mutation of the key conserved residues of the DNase activity abrogates toxicity, indicating that this protein acts as a CDT active subunit [21]. CDT intoxication in *S. typhi* infected cells requires the products of two genes, named *pltB* (persussis-like toxin B) and *pltA* (persussis-like toxin A), and similarly to *cdtB*, their expression is induced after bacteria uptake by the host cells. The role of these components is most likely to transport CdtB from its site of production to the extracellular medium, from where StCDT can also intoxicate cells that have not been infected with the bacterium, in a paracrine manner [22].

## 3. Internalization

CDT is the first bacterial protein toxin known to act in the nucleus of the target cell. As discussed in the previous paragraph, binding to the plasma membrane is a pre-requisite for the intoxication, and as many other bacterial protein toxins, CDT has to cross the plasma membrane to reach the nucleus. The internalization pathway has been mainly studied using HdCDT, AaCDT and EcCDT-II as a model. HdCDT is internalized in HeLa cells by dynamin-dependent endocytosis [23], but it does not require clathrin, since conditional knock down of this protein by RNA interference does not prevent intoxication [16]. Induction of CDT-mediated DNA damage is completely inhibited under conditions that block the fusion of the endosomal compartment with downstream compartments, indicating that the toxin further transits through the endosomal compartment [23]. Using a holotoxin with a modified CdtB subunit, carrying either a sulfation site, or a sulfation and three partially overlapping *N*-linked glycosylation sites at the *C*-terminus, it was possible to demonstrate that the active subunit transits via the trans-Golgi, where sulfation occurs, and it is retrograde translocated via the endoplasmic reticulum (ER), where it is glycosylated [8]. Many ER-translocating toxins exploit the ER-associated degradation (ERAD) pathway to transit from the ER to the cytosol. However, CDT intoxication occurs in cells carrying an altered ERAD system, and its translocation does not require protein unfolding [8,24]. Furthermore, using a combination of confocal microscopy analysis with ER specific markers and biochemical assays, Guerra *et al.* have failed to detect the presence of the CdtB subunit in the cytosol of the intoxicated cells. All these data indicate that most likely the active subunit of CDT is directly translocated from the ER to the nucleus, where it exerts its genotoxic activity [24].

**Figure 1:**
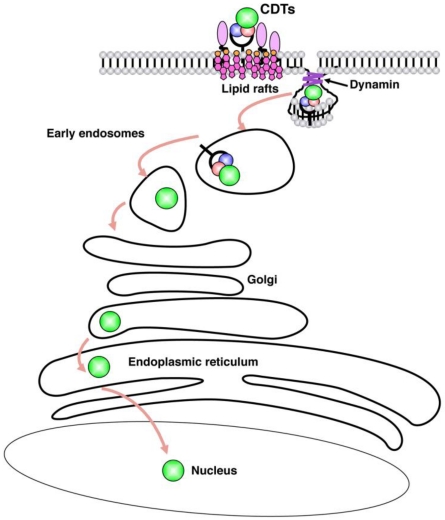
Cytolethal distending toxin (CDT) internalization pathway. Binding of CDT is dependent on the presence of intact lipid rafts, and the toxin is internalized via dynamin-dependent endocytosis into early and late endosomes. The CdtB subunit further transits to the Golgi complex, and is then retrogradely transported to the endoplasmic reticulum (ER). Translocation from the ER does not require the ER-associated degradation (ERAD) pathway, and protein unfolding.

Figure 1 summarizes the key steps in CDT internalization.

How the nuclear translocation of the CdtB subunit takes place is still an open question. Nishikubo and co-workers have identified an atypical nuclear localization signal (NLS) within the amino-terminal region of the AaCdtB, which is essential for nuclear translocation of the recombinant His-tagged CdtB-GFP. Deletion of 11 amino acids within the NLS sequence of CdtB abolishes intoxication [25]. On the other hand, two NLS sequences, designated as NLS1 and NLS2, have been identified in the carboxy-terminal region of the EcCdtB-II. Deletion of these two regions prevents induction of cell cycle arrest and nuclear localization of the holotoxin, without affecting its DNase activity, as tested in *in vitro* assays [26]. Interestingly, deletion of each of these sequences produces a differential localization of the active toxin subunit. Cells intoxicated with a holotoxin containing the EcCdtB-II-ΔNLS1 display a perinuclear distribution, which is consistent with trapping of the active toxin component in the late endosome and/or ER compartment. A diffuse cytoplasmic staining is observed in cells exposed to the EcCdtB-II-ΔNLS2 containing toxin. It is possible that this second NLS may act as scavenger motif to retrieve CdtB molecules, which have escaped to the cytosol, similar to the suggested ER retention function of the KDEL sequence in cholera toxin [27].

The presence of NLSs in entirely different parts of the CdtB molecule in the AaCdtB and EcCdtB-II is still puzzling, considering the high degree of conservation of this subunit amongst different bacteria species [28].

Another important point of discussion is the observation that the majority of the studies on CDT internalization have been performed using recombinant purified soluble holotoxin. However, it has been reported that both EcCDT-II and CjCDT are secreted within outer membrane vesicles (OMVs) [29,30]. It will be interesting to assess whether the delivery of the OMV-associated holotoxin follows the same route as the soluble complex.

## 4. Cellular Responses

### 4.1. Induction of DNA Damage

The first demonstration that CDT is a genotoxin stems from the work of Elwell *et al.* and Lara-Tejero *et al.* who identified position-specific homology between the CdtB subunit from EcCDT-II and CjCDT, respectively, and the mammalian DNase I. The sequence conservation is associated with a functional homology, since preparation of EcCDT-II completely degrades a DNA plasmid substrate in 12 h, and ectopic expression of CjCdtB induces nuclear fragmentation and a marked chromatin disruption in HeLa cells [5,6]. Mutations of the conserved residues required for catalysis or for magnesium binding abolish both the DNase activity as well as the cytotoxic effect.

Direct demonstration of CDT-induced DNA fragmentation was subsequently shown using pulsed field gel electrophoresis (PFGE) analysis both in mammalian cells intoxicated with HdCDT and in budding yeast carrying a conditional CjCdtB expressing plasmid [7,31].

It is noteworthy that the efficiency of CDT to degrade DNA is much lower than that of purified bovine DNase I, as seen both in *in vitro* assays with EcCDT-II and upon microinjection of HdCDT in HeLa cells [32,33].

### 4.2. Activation of DNA Damage Responses

To protect the genome from DNA damage induced by endogenous or exogenous sources, cells activate a series of complex mechanisms. These mechanisms are referred to as DNA damage responses (DDRs) that act to repair the genome and minimize the probability of lethal or permanent genetic damage. The cellular response to DNA damage encompasses multiple repair mechanisms and checkpoint responses that can delay cell cycle progression or modulate DNA replication, and are coordinated primarily by two distinct kinase signaling cascades: the ATM-Chk2 and ATR-Chk1 pathways. The ATM-Chk2 and ATR-Chk1 pathways respond to different types of DNA damage: ATM is recruited to and activated primarily at DNA double-strand breaks (DSBs) in conjunction with the MRE11:RAD50:NBS1 (MRN) sensor complex, while ATR is recruited at sites of single-stranded DNA (ssDNA) in association with its partner protein, ATRIP. Recent evidence indicates that the ATR–Chk1 repair axis is also activated in an ATM-dependent manner in response to DSBs. Prolonged exposure to DNA damage will chronically activate the DDR machinery, including the ATM-Chk2-p53 axis, resulting in either cell death or a long-term cell cycle arrest state known as cellular senescence (reviewed in [34]). This response represents an inducible barrier against acquisition of genomic instability and tumor initiation and/or progression (reviewed in [35]).

Based on the DNase activity of CDT, it is not surprising that intoxicated cells activate the full pattern of DDRs. The cells exposed to CDTs have been shown to arrest in the G1 and/or G2 phases of the cell cycle, or undergo apoptosis, depending on the cell type [36,37,38,39]. The CDT-induced cell cycle arrest resembles the checkpoint response to ionizing radiation (IR), characterized by activation of the ATM kinase and ATM-dependent induction of the tumor suppressor p53 and its transcriptional target, the cyclin-dependent kinase inhibitor p21, phosphorylation of histone H2AX and re-localization of the DNA repair proteins, such as MRE11 and RAD50, to the sites of DNA DSBs [33,39,40,41,42].

Guerra *et al.* recently demonstrated that the proto-oncogene MYC plays an important role in response to CDT- and IR-induced DNA damage, since MYC is required for prompt activation of the ATM-dependent DNA damage pathway in irradiated or intoxicated cells [43].

Normal and cancer cells that survive the acute phase of intoxication by HdCDT possess the hallmarks of cellular senescence. This characteristic phenotype includes persistently activated DNA damage signaling (detected as 53BP1/γH2AX-positive foci), enhanced senescence-associated β-galactosidase activity, expansion of PML nuclear compartments, and expression of IL-6, IL-8, IL-20 and IL-24 [44].

A genome wide analysis performed in budding yeast has identified homologous recombination (HR), activation of the DNA damage checkpoint, and S-phase checkpoint as essential mechanisms for the response to CdtB. This study could not conclusively demonstrate a contribution of the non homologous-end joining (NHEJ) repair pathway to the CdtB-induced DNA lesion. Interestingly, the genes identified in this analysis indicate that there are specific features of the CdtB response that do notfully overlap with the components required for direct DSBs [45]. Conversely, as reviewed above, the majority of the responses activated in mammalian cells fully support a relevant role of the ATM-Chk2 axis in response to CDT-induced DNA damage.

### 4.3. Survival Signals Activated in Intoxicated Cells

The survival of cells with damaged DNA may promote genomic instability and favor tumor initiation and/or progression (reviewed in [46,47]). Characterization of the survival signals in response to CDT intoxication is therefore relevant to understand whether infection with CDT-producing bacteria can contribute to genomic instability and therefore tumor progression.

In adherent cells, irradiation or CDT intoxication is associated with formation of actin stress fibers [48,49]. This effect is regulated by the activation of the small GTPase RhoA, and promotes cell survival in intoxicated cells [31]. Activation of RhoA and actin stress fiber formation in response to CDT is dependent on the RhoA-specific guanine nucleotide exchange factor (GEF) Net1, which is dephosphorylated at a critical inhibitory site [50].

The DNA damage-dependent Net1/RhoA signaling diverges into two different effector cascades: one dependent on the RhoA kinases ROCKI and ROCKII, which controls the formation of actin stress fibers; and one regulated by the mitogen-activated protein kinase (MAPK) p38 and its downstream target MAPK-activated protein kinase 2, which promotes cell survival [50].

The characteristic cell distension observed in intoxicated cells of epithelial and mesenchymal origin is RhoA-independent, and requires a functional PI3-kinase and its downstream effector mTOR [31].

**Figure 2:**
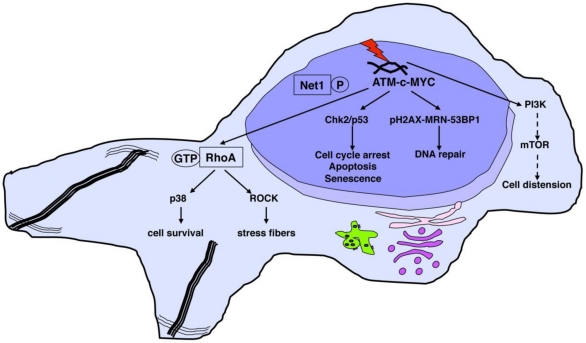
CDT-induced cellular responses. The protein kinase ATM is activated upon CDT-induced DNA damage. c-MYC is required for proper activation of the ATM-dependent DNA damage response, which in turn recruits phosphorylated histone H2AX and the DNA repair proteins, such as the MRN complex, at the sites of DNA strand break. As consequence of the DNA damage checkpoint responses, cells are arrested in the different phases of the cell cycle, and in case of failure to properly repair the DNA damage, they senesce or die by apoptosis. CDT-induced DNA damage promotes dephosphorylation of Net1, and consequent activation of RhoA, which regulates two distinct pathways: (1) induction of actin stress fibers, which requires the RhoA kinases ROCKI and ROCKII; (2) activation of p38 MAPK, associated with a delayed cell death. The characteristic distension observed in epithelial cells is dependent on activation of the PI3-kinase (PI3K) and its downstream effector mTOR.

The cellular responses to CDT are summarized in Figure 2.

To identify novel CDT-induced survival signals, we have screened a yeast deletion library in cells expressing the CdtB active subunit under the control of a galactose-inducible promoter. This screen identified 78 deletion mutants with reduced growth rate following inducible expression of CdtB. Bioinformatics analysis revealed that 12 human orthologs of these genes interacts with the RhoA signaling pathway. Functional studies in mammalian cells showed that *FEN1* (yeast ortholog *RAD27*) promotes RhoA activation, MAPK p38 phosphorylation and ultimately cell survival in response to CDT intoxication [51].

### 4.4. CDT-Induced Apoptosis

While the majority of cells of epithelial and mesenchymal origin are arrested in different phases of the cell cycle, and cell death is a very late event observed after more than 96 h post-intoxication, B and T lymphocytes exposed to HdCDT or AaCDT, respectively, are more susceptible to apoptosis [39,52].

Most of the studies aimed at understanding the molecular mechanisms associated with CDT-induced cell death have been performed using as model AaCDT.

Treatment of activated human T cells with AaCDT induces activation of caspases 8, 9 and 3, and DNA fragmentation is detectable 72–96 h after intoxication [52]. These effects are associated with mitochondrial changes, such as decreased transmembrane potential and elevated levels of reactive oxygen species. Overexpression of Bcl-2 decreases the CDT-induced apoptosis, without inhibiting the G2 arrest [52]. AaCDT intoxication also induces cell death in a biphasic manner in two T cell leukemia cell lines, Jurkat and MOLT-4 [53]. In the presence of the caspase inhibitor z-VAD-fmk, CDT-inducedapoptosis is completely blocked for 16 h in Jurkat cells, suggesting that CDT-induced cell death is dependent on caspase activation. However, a subpopulation of cells dies at a later stage of intoxication (more than 24 h post-intoxication) by a caspase-independent cell death [54].

Also cells of the myeloid linage are sensitive to CDT mediated cell death. AaCDT intoxication induces apoptosis in both proliferating and non-proliferating U937 monocytic cells. The induction of apoptosis in proliferating U937 cells is caspase-dependent and requires the DNase activity of CdtB. In contrast, apoptosis in non-proliferating cells is caspase independent [55]. Immature dendritic cells exposed to HdCDT show signs of cell death 24 h to 48 h after intoxication [33]. Interestingly the effect of HdCDT on DCs is dependent on their stage of differentiation, since LPS-treated DCs are resistant to HdCDT-induced cell death [33]. It is possible that upon DC maturation, the repertoire of surface molecules is modified in such a way that HdCDT is no longer able to bind and be internalized. This hypothesis is supported by the lack of activation of DNA damage sensor complex MRN and stabilization of p53 in LPS-treated DCs exposed to HdCDT [33].

### 4.5. CDT as Phosphatase

Shenker and colleagues have reported that the CdtB subunit from *A. actinomycetemcomitans* exhibits PI-3,4,5-triphosphate (PI-3,4,5-P_3_) phosphatase activity similar to that of the tumor suppressor phosphatases PTEN and SHIP1. Mutation analysis indicates that CDT toxicity correlates with the phosphatase activity, and lymphocytes treated with the toxin exhibit reduced PI-3,4,5-P_3_ levels. Finally, lymphocyte sensitivity to CDT-induced G2 arrest correlates with intracellular levels of PI-3,4,5-P_3_ [56].

Conversely, specific CdtB mutations that inhibit the phosphatase activity but retain the DNase activity, are sufficient to induce cell death in proliferating U937 monocytes [55]. Furthermore, the G2 arrest and cell death induced by conditional expression of CdtB in *Saccharomyces cerevisiae* depend exclusively on its DNase-catalytic residue, since yeast does not harbor the substrate for the CdtB phosphatidylinositol-3,4,5-triphosphate phosphatase activity. These results suggest that the DNA damaging activity alone is sufficient to confer the CdtB toxicity [57].

The discrepancy between the requirements of the different enzymatic activities of CDT may depend on the cell type used as model. It is conceivable that T lymphocytes are more susceptible than all the other cell lines tested to the phosphatase activity of CDT.

## 5. Role of CDT as Virulence Factor

**Figure 3:**
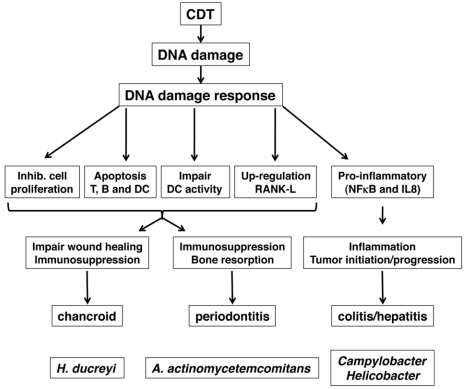
Schematic representation of the possible role of CDT in the pathogenesis of three bacterial diseases: chancroid (*H. ducreyi*), periodontitis (*A. actinomicetemcomitans*), and chronic colitis or hepatitis (*Campylobacter* and *Helicobacter* sp).

In this paragraph, we will briefly review the possible role of CDT in bacterial pathogenesis, and we will focus on three bacterial-associated diseases: inflammatory bowel disease and CDT-producing enteric bacteria; periodontitis and *A. actinomicetemcomitans*; delayed wound healing in chancroid and *H. ducreyi* (Figure 3).

### 5.1. Role of CDT in Colitis and Inflammation-Associated Carcinogenesis

Several CDT-producing bacteria are associated with gastro-enteritis, including *C. jejuni* and *Helicobacter hepaticus*.

In the past ten years, it has been clearly shown that chronic inflammation is associated with enhanced risk of tumor development, and the best-studied model is colorectal carcinoma (CRC) [58,59]. It is likely that in the case of bacterial infection several events, such as the establishment of persistent inflammation, the production of toxins that interfere with regulation of cell cycle progression and apoptosis in association with host genetic factors contribute to the acquisition of genomic instability, and consequently tumor initiation and/or progression. 

Epidemiological evidence has linked chronic bacterial infections with increased risk of tumor development. *Helicobacter pylori* is associated with gastric cancers and has been classified as a type I carcinogen by the World Health Organization [60]. There is growing evidence that other gastric *Helicobacter* species may be associated with chronic liver diseases in humans, including chronic hepatitis, liver carcinoma, chronic cholecystitis, and cholangiocarcinoma [61,62].

To study the role of CDT in chronic infection and inflammation, several animal models have been developed. Adult severe combined immunodeficient (SCID) mice are susceptible to infection by *Campylobacter* sp. To test the role of CDT in the establishment of persistent infection, these mice were infected by intragastric inoculation either with the wild type strain CJ81-176, or with an isogenic strain harboring an inactivating insertion within the *cdtB* gene (Cj81-176 mutant cdtB). Blood, liver and spleen samples were taken 2 h, 6 h and 24 h post-infection (p.i.) to assess for the presence of invasive bacteria. More bacteria-positive samples were detected in mice infected with the wild type CDT producing strain compared to the CdtB deficient *C. jejuni*, indicating that CDT promotes bacteria invasion [63]. However, this was not associated with enhanced levels of intestinal colonization by 7 days p.i. in mice infected with the wild type or the mutant strains [63].

Conversely, gastric colonization of mucin deficient 129/SvJ mice by *C. jejuni* was enhanced if the bacteria produced a functional CDT [64], and *C. jejuni* carrying a wild type CDT were able to colonize the gastro-intestinal tract in 50% of C57BL/129 mice 4 months p.i., while an isogenic strain deficient for CDT expression was not detected in any of the infected mice [65]. Bacterial clearance was dependent on a functional NF-κB complex, since mice homozygously deficient for p50 and heterozygous for p65 (p50^−/−^ p65^+/−^), referred to as 3× mice, showed persistent colonization of stomachs and lower bowels with both *C. jejuni* strains at 2 and 4 months p.i. [65]. 3× mice colonized with the *cdtB* mutant strain had significantly less gastritis and hyperplasia at 4 months post-infection than 3× mice colonized with wild-type *C. jejuni*, indicating that CDT promotes proinflammatory responses [65]. In line with this hypothesis, Hickey and colleagues demonstrated that CjCDT elicits secretion of IL-8, an important mediator of inflammation in INT407 (human embryo intestinal epithelial) cells [66].

CDT is also produced by *H. hepaticus*, an enteropathogenic species, which naturally infects the distal gastrointestinal tract of mice [67]. HhCDT was shown to play a crucial role in persistent bacterial colonization of the small intestine of Swiss Webster mice [68], which was associated with significantly higher production of Th1-associated immunoglobulin G2a (IgG2a), Th2-associated IgG1 and mucosal IgA in the mice infected with wild type *H. hepaticus* when compared to animals exposed to the isogenic strain HhcdtBm7, where the *cdtB* gene was inactivated by transposon mutagenesis [68]. Challenge of C57BL/6 interleukin 10 deficient mice with isogenic *H. hepaticus* mutants revealed that CDT expression is not required for colonization of the murine gut. However, a CDT-negative *H. hepaticus* mutant had a significantly diminished capacity to induce lesions in this murine model of inflammatory bowel disease [69].

Infection of A/JCr mice with wild type or an isogenic mutant of *H. hepaticus* lacking CDT activity induced comparable levels and severity of chronic hepatitis at both 4 and 10 months p.i. The presence of CDT was instead necessary for the development of hepatic dysplasic nodules 10 months after infection. This effect was associated with enhanced hepatic transcription of proinflammatory (*TNF*-*α*, *IFN*-*γ* and *Cox*-*2*, *IL*-*6* and *TGF*-*α*) and anti-apoptotic (*Bcl*-*2* and *Bcl*-*X_L_*) genes, upregulation of hepatic mRNA levels of components of the NF-κB pathway (p65 and p50), and increased hepatocyte proliferation compared with the control or the CDT mutant-infected mice [70].

Collectively, these data demonstrate that CDT is an important virulence factor to promote invasion of enteric bacteria and activate proinflammatory responses that can be associated with chronic inflammation, and its possible progression toward malignant transformation.

### 5.2. Chancroid and Wound Healing

*H. ducreyi* is the causative agent of chancroid, a sexually transmitted disease, characterized by soft and slowly healing genital ulcers (reviewed in [71]). 

Cutaneous wound healing is a complex process involving blood clotting, inflammation, new tissue formation, and tissue remodeling. Neutrophils normally begin arriving at the wound site within minutes of injury, to control bacterial infection and produce proinflammatory cytokines, which probably serve as some of the earliest signals to activate local fibroblasts and keratinocytes. Neutrophils recruitment is followed by accumulation of macrophages, which are essential for effective wound healing, since healing is severely impaired if macrophage infiltration is prevented. Re-epithelialization of the skin requires both migration and rapid proliferation of keratinocytes, fibroblasts and epithelial cells (reviewed in [72]). Adaptive immunity, specifically T lymphocytes are essential for a normal wound healing outcome (reviewed in [73]).

Involvement of CDT in ulcer formation was showed in a rabbit model of chancroid, where intradermal inoculation of *H. ducreyi* co-administered with purified HdCDT resulted in significant aggravation of the bacteria-induced inflammatory lesions and in ulcer development [74].

How can CDT contribute to the delayed would healing observed in chancroid? It has been shown that HdCDT affects cell proliferation and survival of many cell types involved in wound healing, such as primary human fibroblasts, primary keratinocytes and cells of epithelial origin [39,75]. Furthermore, HdCDT may interfere with angiogenesis, since it inhibits proliferation of normal human microvascular endothelial cells from adult dermal tissue (HMVEC-d) and human umbilical vein endothelial cells (HUVEC), preventing new blood vessel formation in an *in vitro* angiogenesis model [76]. 

HdCDT affects also effector cells of the innate and adaptive immune system: intoxication inhibits proliferation and IFN-γ secretion of T lymphocytes and induces apoptosis of B lymphocytes [39,48,77], and monocyte-derived DCs, the key activators of the adaptive immune responses [33,78]. Upon phagocytosis of heat-inactivated *H. ducreyi*, DCs produces the proinflammatory cytokines, IL-1β, IL-6, IL-8, and TNF-α. Preincubation of DCs with purified HdCDT results in an approximate 50% reduction in cytokine secretion [78], suggesting an immuno-inhibitory effect of the toxin.

Targeting DCs may represent a strategy to avoid/delay the onset of immune responses, while inhibition of cellular proliferation may impair the wound healing process, thus increasing pathogen spread from host to host and/or favoring the establishment of a life-long latent infection.

### 5.3. Periodontitis and *A. actinomycetemcomitans*

Periodontitis is a chronic inflammatory disease associated with loss of the supporting connective tissue and alveolar bone around teeth. *A. actinomycetemcomitans* has been described as a member of the indigenous oral microbiota of humans [79].

Does AaCDT contribute to periodontitis? The lack of suitable animal models does not allow answering this question directly. However, AaCDT may interfere with the normal periodontal connective tissue remodeling equilibrium, by over-stimulating osteoclast-dependent bone resorption. Production of mature osteoclasts is regulated by interaction between the receptor activator of NF-κB ligand (RANKL) present on the surface of hematopoietic bone marrow stromal cells, periosteal tissue osteoblasts, as well as T lymphocytes, and its receptor (RANK) expressed on osteclast progenitor cells. This process is negatively regulated by Osteoprotegerin (OPG), which acts as a decoy receptor for RANKL, preventing its interaction with RANK and osteoclast maturation (reviewed in [80]). Therefore, the ratio of RANKL/OPG expression determines the amount of osteoclasts formed and controls the degree of bone resorption. It has been shown that AaCDT is sufficient to induce RANKL expression and downregulate OPG mRNA expression in gingival fibroblasts (GF) and periodontal ligament cells (PDLC), obtained from healthy individuals, and in the T lymphocyte cell line Jurkat [81,82].

Furthermore, AaCDT blocks proliferation and causes apoptosis of mitogen activated human CD8^+^ and CD4^+^ T lymphocytes [37,52,53,54], and peripheral blood mononuclear cells (PBMC) exposed to AaCDT are able to produce a wide range of pro-inflammatory cytokines, such as IL-1β, IL-6, IL-8, and IFN-γ, while no secretion of IL-10, IL-12 and TNF-α was detected [83]. It is conceivable that AaCDT stimulates the innate host immune response, promoting production of a specific set of cytokines, leading to an inflammatory pathology, inhibiting T cell functions and possibly creating a suitable niche for bacterial survival and proliferation.

## 6. Conclusions

In spite of the exponential progress in understanding the CDT mode of action and the cellular responses induced by intoxication, there are still many questions that need to be answered regarding the biology of CDT and its role in disease. We still do not know: (1) how the toxin is translocated from the ER to the nucleus where it exerts its genotoxic activity, and which cellular partners regulate the intracellular trafficking of the toxin; (2) why evolution has positively selected a bacterial genotoxin; (3) what is the contribution of CDT in promoting genomic instability in chronic infections. We are looking forward to an even more exciting and productive period of research in the field of the cytolethal distending toxins.
